# Prevention of tumor seeding during needle biopsy by chemotherapeutic-releasing gelatin sticks

**DOI:** 10.18632/oncotarget.15427

**Published:** 2017-02-16

**Authors:** Ren-Yuan Bai, Verena Staedtke, Xuewei Xia, Gregory J. Riggins

**Affiliations:** ^1^ Department of Neurosurgery and Neurology, Johns Hopkins University School of Medicine, Baltimore, MD, USA; ^2^ Department of Neurosurgery, Affiliated Hospital of Guilin Medical College, Guilin, China

**Keywords:** biopsy, intracranial implantation, tumor seeding, brain tumor, gelatin stick

## Abstract

Needle biopsy is an indispensable diagnostic tool in obtaining tumor tissue for diagnostic examination. Tumor cell seeding in the needle track during percutaneous needle biopsies has been reported for various types of cancers. The mechanical force of the biopsy both directly displaces the malignant cells and causes bleeding and fluid movement that can further disseminate cells. To prevent the risk of tumor cell seeding during biopsy, we developed a gelatin stick loaded with chemotherapeutics such as doxorubicin (DXR) that was inserted into the biopsy canal. The gelatin-doxorubicin sticks (GDSs) were created by passively loading precut gelatin foam strips (Gelfoam) with doxorubicin solution. The dried GDSs were inserted into the needle track through the sheath during the needle biopsy and eventually self-absorbed. We showed that this procedure prevented iatrogenic tumor seeding during needle biopsies in two subcutaneous tumor models. In an alternative application, using GDSs in intracranial brain tumor implantation avoided the outgrowth of tumor from the rodent brain, which could otherwise potentially fuse the tumor with the meninges and distort the results in therapeutic studies in rodent brain tumor models.

## INTRODUCTION

Percutaneous needle biopsy is widely practiced for diagnosis of various cancers, including breast, kidney, liver, head and neck, thyroid, lung, pancreatic cancer and melanoma. In the majority of cases, a biopsy is performed to confirm a putative diagnosis of malignancy. Moreover, with the advent of personalized medicine, obtaining tumor tissue has gained even more importance to optimize treatment decisions.

Various needle devices are currently used and the two main types of biopsies are fine needle aspiration biopsy (FNAB) and core needle biopsy [1, 2]. FNAB utilizes a small-caliber needle, commonly from 22G to 25G, to remove tumor cells by aspiration without preserving the histological architecture of the tissue. A core needle biopsy is performed with a larger hollow needle to withdraw small cylinders of tissue from the suspected tumor. A biopsy needle with an outer sheath (TruCut) is often used in the procedure.

With either biopsy approach, cancer cells that are in general less adherent can detach from the tumor and colonize the surrounding tissue and beyond [3]. Metastasis and/or local invasion initiated by the biopsy procedure can occur in various ways - when the dislodged cancer cells enter the blood or lymphatic circulation, or loose cells left in the needle track by the retracting needle or displaced cells move with fluid pressure up the needle track [4].

The frequency and significance of tumor seeding associated with needle biopsies in various cancers remain largely controversial in spite of numerous surveys and studies. A number of early studies may have greatly underestimated the seeding rate as they were based on patient and physician reporting, without verification by an active cross-sectional imaging or histological analysis [5]. Biopsies of breast cancer appeared to be most prone to needle track tumor seeding, with up to 22 % of the patients affected in seven studies in which needle tracks underwent histological analysis following surgical excision shortly after the biopsy [4]. It is important to note that not all tumor cells initially seeded along the needle track will result in metastasis because dislodged tumor cells will have to escape immune surveillance and other defense mechanisms in order to assure survival and local expansion. In addition, tumor seeding have been reported with percutaneous needle biopsies in lung, liver, renal, head and neck cancers but with a lesser frequencies [3, 4].

Likewise, in preclinical animal studies tumor seeding also poses a significant challenge, mainly resulted from the tumor implantation procedure itself. Excessive tumor seeding into the surrounding can potentially distort the results of therapeutic studies and affect the reproducibility in unpredictable ways. One illustrative example is the intracranial implantation of brain tumor cells in rodents, which has been a very useful tool for studying brain tumor biology and therapeutic development [6]. Blood-brain barrier (BBB) is a critical limitation restricting the majority of cancer therapeutics from reaching the brain tumor [7]. We observed that in intracranial rodent brain tumor models, malignant tumors often grow along the needle track up to the burr hole and fuse with the meninges. This growth along the needle tract could create a different microenvironment that results in a more aggressive growth pattern,shorter survival and altered response to certain therapies. Hence, in this study we attempted to reduce the risk of tumor cell seeding of various preclinical animal models *via* development and insertion of chemotherapeutic-loaded gelatin sticks into the needle track.

## RESULTS

### Creating doxorubicin-loaded gelatin sticks for implantation

**Figure 1:**
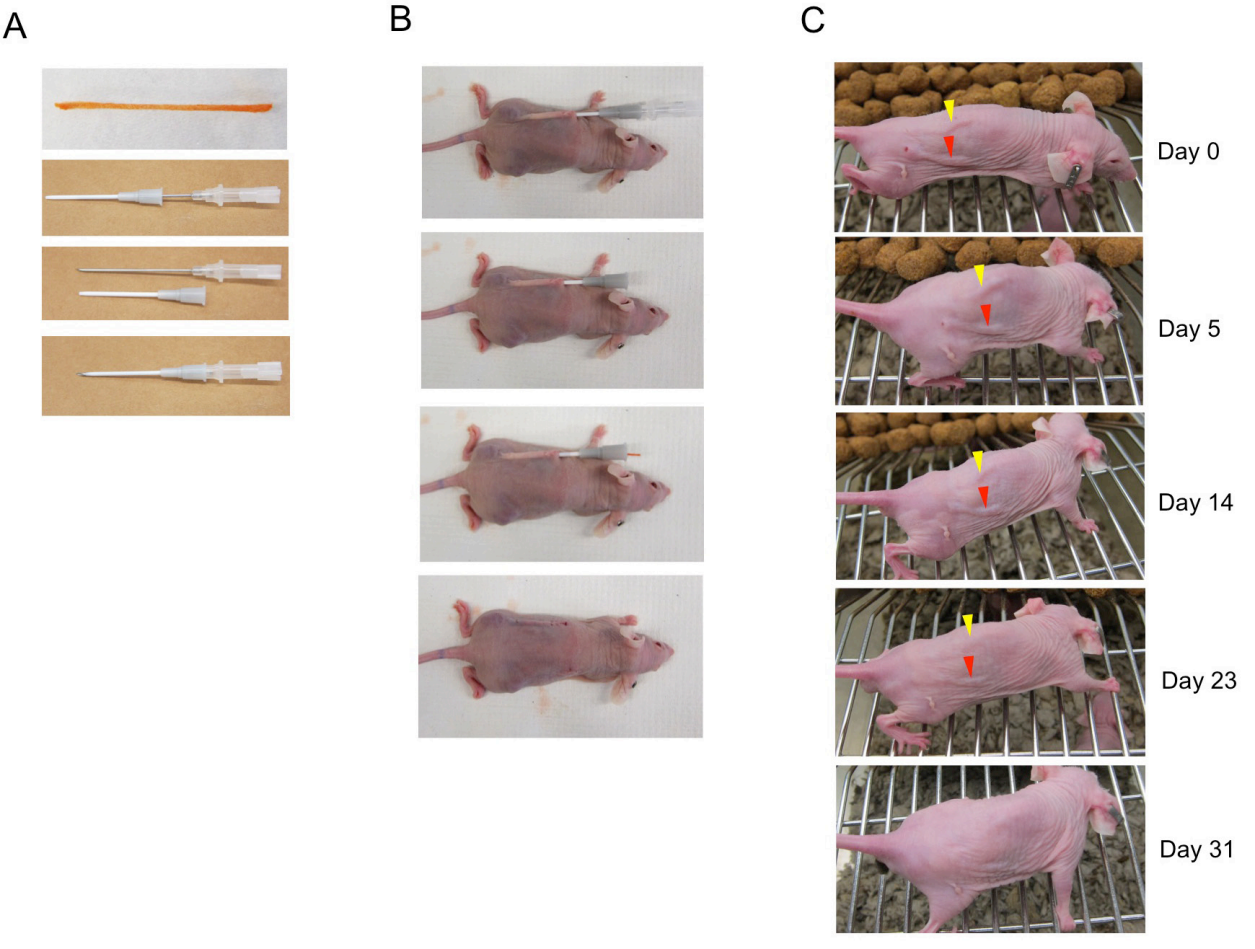
Gelatin sticks supplemented with doxorubicin (DXR) are inert in subcutaneous applications **A**. Preparing gelatin-doxorubicin stick (GDS). A gelatin stick was made by soaking and rolling a 20×3x1 mm Gelform^®^ sponge in doxorubicin solution and left drying overnight. A 16 gauge needle with sheath is illustrated as applicator. **B**. A GDS was inserted subcutaneously in an athymic nude mouse. **C**. GDSs made by 100 μg/ml (yellow arrowhead) or 500 μg/ml (red arrowhead) doxorubicin solution were implanted subcutaneously in a athymic nude mouse and evaluated for over a month for skin appearance. Both GDSs were absorbed after 31 days without obvious skin damage. Experiment was done in five mice.

Gelfoam compressed sponge is a medical product intended as a hemostatic for bleeding surfaces during surgery. The commercially purchased gelfoams were cut into desired sizes, soaked in doxorubicin solutions, rolled and dried at 4°C as described in the Materials and Methods. Doxorubicin was chosen as a representative of chemotherapeutics because it demonstrated relatively low IC_50_ with the selected cancer cell lines compared to other commonly used chemotherapeutics including paclitaxel, topotecan, CPT-11, docetaxel, carboplatin and temozolomide, which have been tested in our previous study [8]. After saturation with doxorubicin of various concentrations, the dried GDSs were 20-22 mm long and 1 mm wide (Figure 1A upper panel), and were stored at -20°C. GDSs were rigid and could pass a sheath of a 16G needle as shown in Figure 1A. In a subcutaneous implantation of GDS, a 16G needle with a sheath was first inserted under the skin of an athymic nude mouse that carried the respective subcutaneous tumor. After tissue collection and withdrawal of the needle, a GDS was inserted through the sheath and pushed to the end by the needle while the sheath was retracted, with the GDS remaining in the skin (Figure 1B). To evaluate the potential subcutaneous toxicity, two different CDSs, made of 250 (yellow arrowhead) or 500 μg/ml (red arrowhead) doxorubicin, were implanted in athymic nude mice (Figure 1B). Over a course of a month, no subcutaneous toxicity was observed in a group of five mice and the skin irregularity caused by the initial GDS implantation gradually disappeared.

### GDSs prevented tumor seeding in needle biopsy of tumors in mice

**Figure 2:**
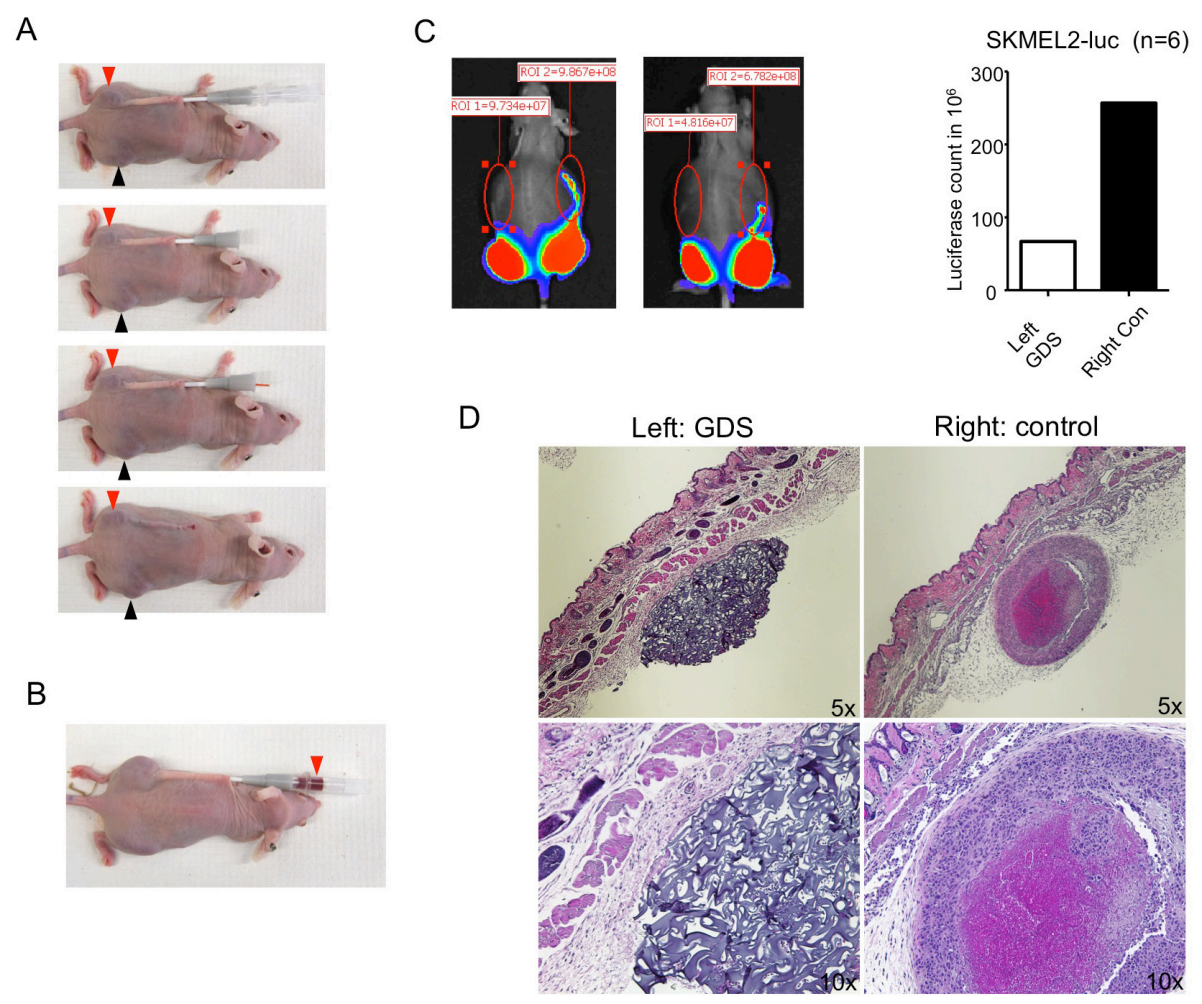
GDS prevented tumor seeding in biopsy of subcutaneous SKMEL2 tumor **A**. A simulation of core needle biopsy was performed with a 16G needle with sheath into the subcutaneous SKMEL2 tumor on the left side of the mouse using a 16G needle with sheath and a GDS was implanted in the needle track. Similar procedure was done to tumor on the right side without GDS implantation (not shown). Red and black arrowheads: SKMEL2 tumors. **B**. An example of extensive bleeding (red arrowhead) during the core needle biopsy of SKMEL2 tumor. **C**. Ten days after the biopsy, tumor seeding was monitored by luciferase activity *via* Xenogen. Two mice were shown as example (right pictures). **D**. Transverse sections of subcutaneous GDS and seeding tumor resulted from needle biopsy. SKMEL2 human melanoma cells expressing luciferase were grown subcutaneously in athymic nude mice and biopsy was performed by inserting a 16G needle with sheath subcutaneously as shown in Figure 1B.

Tumor cell seeding after percutaneous needle biopsies can occur in various types of cancers [5, 9, 10]. In this study, we studied subcutaneously grown SKMEL2, a highly aggressive human melanoma xenograft, to test tumor seeding after core needle biopsy and the efficacy of GDS. We used a 16G needle with a coaxial sheath to simulate the coaxial core needle biopsy. SKMEL2 cells were transfected with luciferase to monitor tumor growth. A 16G needle with sheath was inserted underneath the skin, running 20-25 mm before penetrating into the tumor core. After retraction of the needle, a GDS saturated with 250 μg/ml doxorubicin was implanted (Figure 1A). Penetration of the needle into the tumor caused tumor bleeding, with the blood running along the sheath to subsequently fill the needle track. Figure 1B illustrates an occurrence of this extensive bleeding. Both the tip of the sheath and the blood from tumor vasculatures potentially become sources of tumor cell seeding in the needle track. Ten days after the biopsy, tumor cell seeding was evaluated by Xenogen. An evaluation of the untreated controls revealed tumor cell seeding along the needle track, while the needle track implanted with GDS remained largely tumor free (Figure 2C). Subsequently, mice were euthanized and transverse sections of the skin samples showed the microscopic appearance of subcutaneous GDS and the tumor formation in the needle track (Figure 2D).

**Figure 3:**
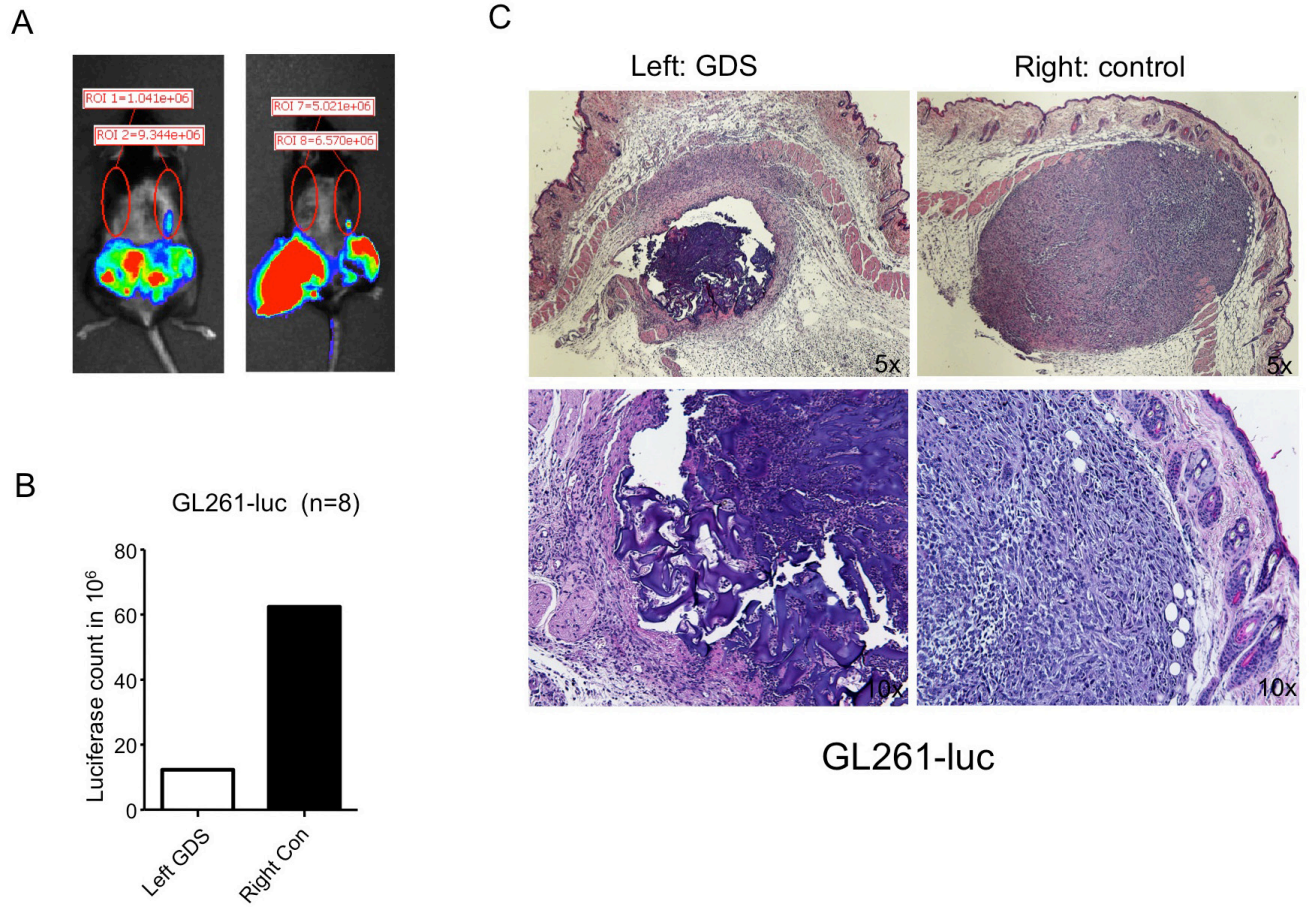
GDS prevented tumor seeding in biopsy of subcutaneous GL261 tumor **A**. and **B**. GL261 mouse glioma cells expressing luciferase were grown subcutaneously in C57BL6 mice and a simulation of core needle biopsy was performed by inserting a 16G needle with sheath subcutaneously as shown in Figure 2A. A GDS was implanted in the needle track on the left side and no GDS was implanted to the right side as control. Ten days after the biopsy, tumor seeding was monitored by luciferase activity *via* Xenogen. **C**. Transverse sections of subcutaneous GDS and seeding tumor resulted from needle biopsy.

The same procedure was performed in C57BL6 mice implanted subcutaneously with syngeneic GL261 glioma cells. Similarly, ten days after the needle biopsy, the control side showed increased incidents of tumor seeding in the needle tracks, as reflected by the luciferase signals, in comparison to the contralateral side implanted with GDS (Figure 3A & 3B). H&E staining of the skin sections confirmed the tumor growth in the needle track on the control side (Figure 3C, right panels).

### Using GDSs in intracranial implantation of brain tumor cells

**Figure 4:**
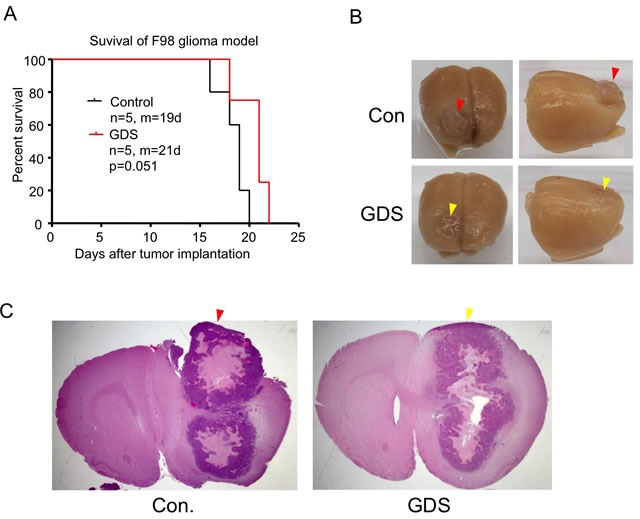
GDS prevented meningeal growth of brain tumor implantation **A**. F98 rat glioma cells were implanted intracranially in F344 rats without (control) or with a 1-mm GDS. Survival curved of rats were compared and showed marginal but insignificant difference (*P* = 0.051). m: median survival in days after implantation. **B**. Marcoscopic appearance of rat brains implanted with F98 glioma without (−) or with (+) GDS. In the brain without GDS, Tumor outgrew around the burr hole (red arrowhead). **C**. Coronal sections of rat brains implanted with F98 glioma without or with GDS (H&E staining).

Intracranial implantation in rodents is a highly useful tool in brain tumor research and therapeutic development. It has been observed that certain implanted brain tumors could grow along the needle track towards the burr hole and end up fusing with the meninges that are not restricted by the BBB. This could potentially distort the results of therapeutic assessment in brain tumor models. Similar to the seeding of tumor cells in needle biopsy, aside from the risk of dragging tumor cells along the needle track during retraction of the implantation needle, bleeding caused by the needle could fill the needle track with tumor cells-containing blood all the way to the meninges and burr hole. In this study, we examined GDS in preventing this outgrowth of implanted brain tumors. Inserting a GDS as short as 1 mm into the burr hole without directly penetrating into the brain tissue mechanically did not significantly alter the survival of syngeneic F98 rat glioma model (Figure 4A). In the control group, the fully grown F98 tumor protruded outside the brain surface (Figure 4B & 4C) and grew adjacent or possibly attached to the meninges. In contrast, the brain tumor implanted with GDS did not spread out of the brain surface to the meninges even up to the point where the tumor resulted in the animal's death (Figure 4B & 4C).

## DISCUSSION

Gelatin is a mixture of proteins and peptides obtained by partial hydrolysis of collagen from the cartilage, skin and bones of animals. Gelatin products are safe for human consumption and medical applications. For example, Gelfoam compressed sponge is produced as a hemostatic for bleeding surfaces and is frequently used in dentistry and surgery. Our choice of Gelfoam was based on its ease of handling and loading capacity of chemotherapeutics. Gelatin is most commonly available as powder, which, due to its thermoplastic properties, can be dissolved in aqueous solutions by heating and is hardened upon cooling and drying. It is also conceivable to produce GDS directly from the gelatin powder and doxorubicin solution using heating, cooling and drying. However, this will require more biomaterial development to produce standardized GDS fitting to the sheath of a biopsy needle of 16G or thinner. In this study, we have shown that doxorubicin-loaded gelatin sticks prevented tumor seeding in needle tracks in two different mouse tumor models, while being safe to use and causing no skin irritation in the usually sensitive athymic nude mice.

The risk of tumor seeding through biopsy or aspiration is a well-recognized problem that can occur even when meticulous caution is applied. Over the years, several technical improvements have been introduced in the clinical practice to minimize this risk, including cryoablation and coaxial cutting needle technique. Percutaneous cryoablation guided by imaging is a minimally invasive biopsy procedure with a lower risk of needle-track seeding, which involves a two-step freezing method to kill tissue around the biopsy-needle sheath to avoid needle-track seeding [11, 12]. Coaxial cutting needle technique is used in the core needle biopsy that applies a needle introducer that remains in position during multiple cutting needle sampling, which may protect the needle track from tumor seeding [3]. In this study, we used the needle with a sheath that simulated the coaxial cutting needle technique. Despite the protection of the needle introducer/sheath, excessive bleeding caused by penetrating and sampling the tumor tissues can fill the blood into the needle track and potentially seed dislodged tumor cells. In addition, detached tumor cells attached to the sheath can be left behind in the needle track. We further demonstrated that implanting GDS could prevent such seeding in the rodent flank tumor models. It is noteworthy that percutaneous biopsies of organs such as kidney, lung and liver entail penetration of internal organs and body cavities that can trap loose tumor cells outside the needle tracks. Thus, it is feasible that a combination of cryoablation, coaxial cutting needle and GDS implantation could minimize the risk of tumor seeding.

Lastly, reflux of tumor cell-containing fluid out of intended implantation site could complicate intracranial tumor models [13]. Indeed, we observed the outgrowth of a subset of brain tumors through the burr hole after performing stereotactic brain tumor implantations in laboratory animals. For instance, syngeneic GL261 glioma-bearing C57BL6 mice typically do not form an outgrowth and it is noted that the application of GDS in mouse models is less feasible due to small size of the mouse skull. By contrast, syngeneic F98 and 9L rat gliomas and also VX2 tumor cells that can be intracranially implanted into New Zealand White rabbits frequently extent beyond the brain surface, where it can form another mass or potentially fuse with the meninges. This is an important problem to recognize because such a scenario could influence the results of therapeutic studies in unexpected ways and impair reproducibility of data. To prevent this from happening, GDS could be utilized by inserting an only 1mm long filament into the burr hole where it remained between the bone and meninges, but presumably did not mechanically penetrate into the brain tissue. Although GDS did not significantly affect the animal survival in our study, we have not assessed the possibility of it interfering with any tested drugs.

In conclusion, we created an absorbable gelatin filament loaded with chemotherapeutics that, when left in the needle track after biopsy or other surgical procedures, may prevent iatrogenic tumor seeding.

## MATERIALS AND METHODS

### Cell lines and tissue culture

The human melanoma cell line SKMEL2 was obtained from ATCC. Mouse glioma cell line GL261 expressing luciferase was described before [14]. All cells were maintained in DMEM media supplemented with 10% fetal bovine serum and antibiotics. Cells were kept in frozen stocks upon reception and were not additionally authenticated. Tissue culture was maintained at 37°C in humidified air containing 5% CO2.

### Luciferase expression by lentivirus

Lunciferase expression was previously described [15]. Briefly, Firefly luciferase cDNA from pGL3-basic (Promega, Madison, WI) was subcloned in pFUGW and transfected along with CMVΔR8.91 and pMD.G in 293T cells by Lipofectamine 2000 (Invitrogen, Carlsbad, CA). Virus was harvested after 48 hours and SMKEL2 cells were infected by incubating with 8 μg/ml polybrene (Sigma, St. Louis, MO) in the growth medium.

### Animal experiments

All animal works were approved by the Animal Care and Use Committee (ACUC) of the Johns Hopkins University.

Female athymic nude mice or C57BL6 mice, 5-6 weeks of age, were purchased from National Cancer Institute (Frederick, MD). For the implantation procedure, female athymic nude mice were anesthetized *via* intraperitoneal injection of 60 μl of a stock solution containing ketamine hydrochloride (75 mg/kg) (100 mg/mL; Ketamine HCl; Abbot Laboratories, Chicago, IL, USA) and xylazine (7.5 mg/kg) (100 mg/mL; Xyla-ject^®^; Phoenix Pharmaceutical, St. Joseph, MO, USA) in a sterile 0.9% NaCl solution. 5×10^6^ GL261 or SKMEL2 cells in 100 μl expressing luciferase were mixed with equal volume of Matrigel (BD Bioscience) and injected subcutaneously in the flanks of mice.

Luciferase activity was determined by a Xenogen instrument (IVIS 200) with intraperitoneal injection of 2 mg/mouse D-luciferin potassium salt solution (Gold Biotechnology, St. Louis, MO). After 15 min following the injection, the animals were scanned for 1 min at a distance of 20 cm.

### Making and application of gelatin-doxorubicin sticks (GDSs)

Gelfoam^®^ absorbable gelatin sponges (2 × 2 cm) were manufactured by Pfizer. A piece of 20 × 4 x 1 mm was cut out from the sponge by a scalpel and soaked in doxorubicin hydrochloride (DXR) solution for two minutes. The gelatin piece was then rolled in the form of a stick, straightened, air-dried on a Petri dish over night at 4°C and stored at -20°C.

Mice were anesthetized first and a 16G catheter needle (Jelco, No. 4042) was inserted under the skin for about 20 mm. For mice with subcutaneous tumors, the needle penetrated into the tumor body and rotated a round in order to displace sufficient amount of tumor cells. The needle was then retracted out of the skin with the sheath being left inside. At this point, a 20 mm GDS was inserted through the sheath by a fresh 16G needle and left inside after removal of the sheath. For the tumor on the control side, no GDS was inserted.

For brain tumor implantation in rats, female F344 Fisher rats (weight 100-150 gram) were purchased from the NCI. Rats were anesthetized *via* intraperitoneal (i.p) injection composed of ketamine hydrochloride (75 mg/kg; 100 mg/mL; ketamine HCl; Abbot Laboratories) and xylazine (7.5 mg/kg; 100 mg/mL; Xyla-ject; Phoenix Pharmaceutical) in a sterile 0.9% NaCl solution. Subsequently, rat F98 glioma cells at 20,000 cells/μl were loaded in a 24G Hamilton syringe needle (7105KH) and the needle tip was cleaned from tumor cells with an ethanol wipe. The needle was inserted stereotactically into the burr hole located 3 mm lateral and 2 mm anterior to the bregma in the depth of 6 mm. After a 1 min pause, the needle was retracted by 1 mm and 1 μl of cells were injected slowly over 1 min. After pausing for 5 min, the needle was slowly removed. For the implantation with GDS, a piece of 1 mm GDS created with 50 μg/ml doxorubicin was inserted in the burr hole. No GDS was used in the control rats. The burr hole was sealed by bone wax and the skull was irrigated by 0.5 ml sterile PBS.
